# Therapeutic Strategies for COVID-19 Lung Disease in Children

**DOI:** 10.3389/fped.2022.829521

**Published:** 2022-03-07

**Authors:** Elisabetta Gatti, Marta Piotto, Mara Lelii, Mariacarola Pensabene, Barbara Madini, Lucia Cerrato, Vittoria Hassan, Stefano Aliberti, Samantha Bosis, Paola Marchisio, Maria Francesca Patria

**Affiliations:** ^1^Università Degli Studi di Milano, Milan, Italy; ^2^Fondazione IRCCS Cà Granda Ospedale Maggiore Policlinico, Milan, Italy; ^3^Department of Biomedical Sciences, Humanitas University, Via Rita Levi Montalcini 4, Pieve Emanuele, Milan, Italy; ^4^Respiratory Unit, IRCCS Humanitas Research Hospital, Via Manzoni 56, Milan, Italy

**Keywords:** COVID-19, children, respiratory support, immunomodulant treatment, antiviral therapy, lung disease

## Abstract

The novel Severe Acute Respiratory Syndrome Coronavirus 2 (SARS-CoV-2) infection has milder presentation in children than in adults, mostly requiring only supportive therapy. The immunopathogenic course of COVID-19 can be divided in two distinct but overlapping phases: the first triggered by the virus itself and the second one by the host immune response (cytokine storm). Respiratory failure or systemic involvement as Multisystem Inflammatory Syndrome in Children (MIS-C) requiring intensive care are described only in a small portion of infected children. Less severe lung injury in children could be explained by qualitative and quantitative differences in age-related immune response. Evidence on the best therapeutic approach for COVID-19 lung disease in children is lacking. Currently, the approach is mainly conservative and based on supportive therapy. However, in hospitalized children with critical illness and worsening lung function, antiviral therapy with remdesivir and immunomodulant treatment could be considered the “therapeutic pillars.”

## Background

Currently, there is no evidence on the best therapeutic approach for COVID-19 lung disease in children and indications are mostly derived from clinical trials conducted in adults. Our manuscript provides guidance for the management of SARS-CoV-2 infection in children.

## Search Strategy and Selection Criteria

References for this review were identified through searches on PubMED from 1 December 2019 to 23 June 2021 by using the following search strategy: “COVID-19 treatment in children,” filter age 0–18. One hundred and thirty-two papers were identified. Reference lists of the articles identified by this search strategy were also searched. Only articles in English were included in this review.

## SARS-CoV-2 Infection in Children

The novel Severe Acute Respiratory Syndrome Coronavirus 2 (SARS-CoV-2), responsible for the COVID-19 pandemic, belongs to the family of “Coronaviridae” which includes other viruses, called Human Coronavirus (HCoVs), mostly causing respiratory and gastrointestinal disease, common in pediatric age (1). While respiratory viral infections are, in general, more frequent and severe in children than in adults, SARS-CoV-2 differs for relative sparing of pediatric population. Particularly, the infection rate appears to be similar between children and adults, but children develop a milder illness with a low case fatality rate (1, 2). According to a study by the Chinese Center for Disease Control and Prevention conducted on nearly 45.000 adults with COVID-19, severe disease with lung involvement was observed in 14% of case (3, 4), while Acute Respiratory Distress Syndrome (ARDS) developed in 42% of cases presenting with COVID-19 pneumonia (5). In contrast, Pediatric ARDS (PARDS) has only been described in a small number of cases (6). One of the largest pediatric reviews including 2,135 Chinese pediatric patients with COVID-19 reported that 5.8% of children had severe or critical respiratory disease, 0.6% developed ARDS and only one died (7). The reasons for mild COVID-19 disease in children remain elusive (8). It is hypothesized that children may have a different expression of Angiotensin Converting Enzyme (ACE) 2 receptor in alveolar type 2 cells (8–11), cross-immunity with other HCoVs or greater activity of the innate and adaptive immune system (IS) (12, 13). Finally, the lack of a variety of comorbid conditions common in adults, such as arterial hypertension, heart disease and diabetes mellitus, contributes to better outcome in pediatric age. Even children with chronic lung disease, such as asthma and Cystic Fibrosis (CF), are relatively spared from severe SARS-CoV-2 infection, although they are known to be at increased risk of developing severe complications during other viral infections (14, 15). For example COVID-19 is a possible cause of asthma exacerbations (16) but asthma does not appear to be a risk factor for hospitalization due to COVID-19 (17). In fact, incidence of asthma in children hospitalized for COVID-19 seems to be lower compared to general pediatric population (18). However, the Center for Disease Control and Prevention in the United States (19) and the European Academy of Allergy and Clinical Immunology (20), on a more observational basis rather than scientific evidence, consider children with moderate-to-severe or uncontrolled asthma to be at increased risk of developing more severe COVID-19. For the same reason patients with Cystic Fibrosis (CF) should be considered at risk of developing severe manifestations with COVID-19 (21), even if, according to Italian and European data, the incidence of SARS-CoV-2 infection among CF population (0.07%) appears to be lower compared to the general population (0.15%) (21, 22). The apparent lower rate infection in CF may be partly attributable to the lower average age of the CF population and to the adoption of shielding and protective self-isolation (21). By contrast, bronchodysplasia (BDP) seems to be related to an increased risk of severe infection (15). Although Moeller et al. (15) reported only 9 cases of bronchodysplasic children with COVID-19 in their survey including 174 centers, all of them required targeted therapies.

## Clinical Phenotypes

Five clinical patterns of SARS-CoV-2 are recognizable in children (2, 7, 12): more than half has asymptomatic or mild presentation (fever, asthenia, myalgia, and cough); about one third has moderate symptoms (mild respiratory distress); 5% develops more severe respiratory symptoms (dyspnoea, hypoxia) and <4% requires intensive care due to acute respiratory failure or, sometimes, in the context of Multisystem Inflammatory Syndrome in Children (MIS-C) (12, 23–25). Lung imaging performed in children with mild phenotype does not show any pathognomonic sign. Nevertheless, radiologic abnormalities are frequent in the moderate/severe phenotype, including airspace opacities, interstitial alterations and subpleural ground glass lesions; consolidations with surrounding “halo sign” were reported in more than 50% of CT findings in this type of patients (26). Little is known about the sequelae of SARS-CoV-2 infection in children. Lung ultrasound findings and pulmonary function were evaluated in children after an asymptomatic or mildly symptomatic SARS-CoV-2 infection, but no pulmonary complications were detected. A longer follow up time is needed (27).

## Pathogenesis of SARS-CoV-2 Infection in Children

**Figure 1:**
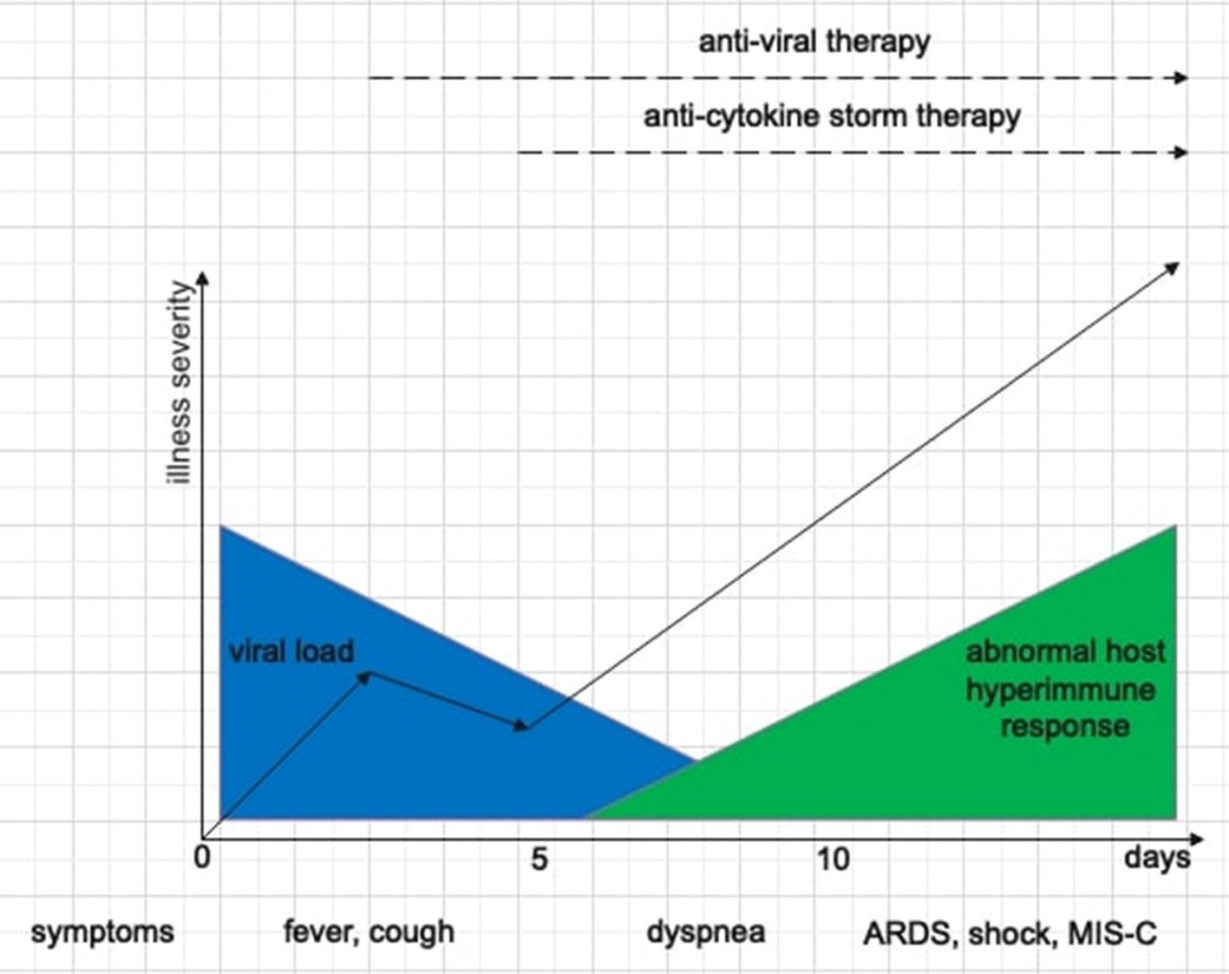
Schematic representation of the two phases of SARS-CoV-2 disease, the correlation between clinical features, SARS-CoV-2 disease stages, and therapeutic options.

The pathogenesis of SARS-CoV-2 infection is characterized by two distinct but overlapping phases (Figure 1). The first one, triggered by SARS-CoV-2 itself, depends on the viral load and the clearance capacity and is responsible for the initial respiratory symptoms. This phase ends with the elimination of the virus or the progression of lung disease. Sometimes, a second phase, triggered by host immune response and characterized by “cytokine storm,” may occur. While hyperinflammation in adult COVID-19 patients is associated with respiratory failure in the setting of ARDS, in children the predominant manifestation is MIS-C, with relative lung sparing (3, 12, 24). The age-related lung involvement could be explained by different immunological mechanisms behind hyperinflammation in children and adults (28). Diorio et al. (24) performed a systematic prospective evaluation of clinical and laboratory biomarkers (cytokine profiles, measures of viral burden, markers of vascular damage) in children who presented minimal COVID-19, severe COVID-19 and MIS-C. The positive but high SARS-CoV-2 RT-PCR cycle threshold from nasopharyngeal aspirates (which inversely correlates with viral burden) associated with MIS-C supported a postinfectious etiology for this phenomenon that was previously postulated but not demonstrated. Other clinical markers also differed between the groups, including markers of vascular injury (D-dimers and B-natriuretic-proteins) and cytokines profile (IL-6, IFN-γ, IL-10, TNF-α). Furthermore, Diorio et al. (24) postulated that immune activation related to SARS-CoV-2 was associated with endothelial dysfunction, and they measured soluble-C5b-9 (sC5b-9), a product of the terminal complement cascade associated with microangiopathy in numerous settings. sC5b-9 was significantly elevated in patients with severe COVID-19 compared to those with minimal COVID-19 and MIS-C, supporting experimental therapeutic strategies against complement (29). Finally, also qualitative and quantitative differences in the host immune response to SARS-CoV-2 infection have been studied to understand the reason of relative lung sparing in children. Analysis of antibody response showed a less productive infection and/or a better viral clearance in children compared to adults with greater neutralizing antibodies power, indicative of a more severe primary lung infection (12).

## Lung Disease Treatment

Understanding the pathophysiological mechanisms of SARS-CoV-2 disease is essential to develop therapeutic strategies: in the acute infectious phase the goal is the elimination of the virus using antiviral drugs, while in the post-infectious phase immunomodulating drugs mitigate the “drift” of immune response. Therapeutic approach depends on disease severity (Table 1). In children with mild phenotype, no specific therapy is required, except for antipyretics [ibuprofen orally 5–10 mg/kg or paracetamol orally 10–15 mg/kg (30)] in case of fever >38.5°C. Despite initial warning, a harmful effect of ibuprofen in patients with COVID-19 has not been established (31–33). Moderate phenotypes require supportive care, such as intravenous (IV) rehydration and respiratory support. Severe/critical phenotypes require personalized therapeutic approach, including the use of second-line drugs such as antivirals, immunomodulators and a stronger respiratory support. As in other viral respiratory infections, bronchodilators and/or inhaled steroids are needed in case of bronchospasm (25). Concerning asthmatic children, optimal disease control is the first goal to protect these patients from a severe course of COVID-19, therefore therapies with antihistamines, corticosteroids, leukotriene-receptor-antagonists and bronchodilators should not be stopped (11). Instead, patients with severe asthma using biologicals (omalizumab, mepolizumab, dupilumab), should stop the treatment in case of an active SARS-CoV-2 infection. Due to their long half-life in the range of a few weeks, the impact of their suspension on acute management and what is the risk of losing disease control might be remain unclear (34). In addition, the use of pressurized metered-dose inhaler via a spacer device is preferred to nebulizers, which should be avoided due to the increased risk of viral dissemination.

**Table 1:** Treatment of pediatric SARS-CoV-2 pulmonary disease.

**Clinical phenotype**	**Antiviral therapy**	**Immunomodulating therapy**	**Supportive therapy**
**Asymptomatic infection**	–	–	–
**Mild**	–	–	Antipyretics
Fever, upper respiratory signs. *No respiratory distress*			
**Moderate** Fever, asthenia, lower respiratory signs *Mild respiratory distress*	Remdesivir §	–	If SpO_2_ <95%: low-flow oxygen therapy (nasal cannula, facial mask, facial mask with Venturi system)
**Severe** Tachypnea Hypoxemia (SpO_2_ < 92%) Feeding difficulties, lethargy *Severe respiratory distress*	Remdesivir	Methylprednisolone iv/ oral dexamethasone	HFNC → NIV (CPAP or NIV *bi-level*) VTE # Antibiotics #
**Critical** PARDS (acute respiratory failure + bilateral pulmonary opacities *+ PaO_2_/FiO_2_ <300)	Remdesivir Casirivimab/Imdevimab (> 12 years)	Methylprednisolone iv Anakinra, ocilizumab	NIV (CPAP or NIV *bi-level*) → Invasive ventilation, prone positioning VTE # Antibiotics #

(^*^) in differential diagnosis exclude heart failure or fluid overload; (§) in selected cases: severe comorbidities with high risk of negative evolution; (#) at clinical discretion. HFNC, high-flow nasal oxygen; NIV, Non-invasive Ventilation; PaO2, arterial partial pressure of oxygen; FiO2, inspired fraction of oxygen.

### Antiviral Therapy

Currently, no specific antivirals have proven efficacy and safety in children with COVID-19. At the beginning of the pandemic, oseltamivir, a neuraminidase inhibitor, was used to treat patients with early diagnosis but it was found to be ineffective because coronaviruses do not produce neuraminidase (30, 35). Equally ribavirin, a nucleotide analog able to inhibit viral RNA polymerase, was initially used in children with COVID-19, but it was no longer confirmed (30, 36). Interferons are critical proteins involved in immune activation and regulation, with antiviral properties. A consensus of Chinese experts suggested the use of inhaled interferon-alpha, but the effectiveness of this therapy has not been demonstrated yet (30, 37–39). Hydroxychloroquine, thanks to its antiviral and immunomodulatory activity, was thought to be a potential agent to treat SARS-CoV-2 infection. However, considering the lack of evidence and the risk of side effects (retinal toxicity, QT-interval prolongation), hydroxychloroquine was no longer recommended (32, 35, 36, 40, 41). Other broad-spectrum antivirals such as lopinavir/ritonavir (LPV/r) and remdesivir (RDV) have been tested in COVID-19 treatment (32) and available data derive from clinical trials in adults (42–44) or single pediatric cases (42). Lopinavir, a protease inhibitor, was used in combination with ritonavir, which inhibits CYP-3A-mediated metabolism of lopinavir. Cao et al. (32, 45) in a randomized-controlled trial, reported that no benefit was associated with LPV/r treatment in hospitalized adults. Moreover the use of LPV/r was also found to increase the risk of liver injury (46). Thus, the use of LPV/r is no longer recommended (36). Chiotos et al. (36) in their multicenter guidance suggested RDV as the preferred antiviral agent. RDV is a nucleoside analog able to bind viral RNA polymerase. It was developed in 2017 for Ebola and *in vitro* studies showed its effectiveness against different CoVs (25, 47). Since May 2020, the FDA approved the administration of RDV in hospitalized children and adults suffering from suspected or laboratory-confirmed severe/critical COVID-19 (25, 42, 47). Subsequently, the indication was extended to hospitalized pediatric patients with moderate disease, based on clinical judgement (36). However, recent trials showed no impact of RDV in the treatment of COVID-19 (35). Thus, RDV should be considered only for children with high care complexity and comorbidities (e.g., BDP), possibly at early course of the illness (42). The dosage indicated in pediatric age (<40 kg) is 5 mg/kg IV on the first day, followed by 2.5 mg/kg/day IV; in critical cases minimum duration therapy of 5 days can be extended up to 10 days (36). These recommendations are based on adult physiologically based pharmacokinetic modeling and reflect those used in the Ebola trials, as well as those recommended “compassionate use” program. While these doses are expected to provide similar drug exposure to those observed in healthy adults, there are no published pharmacokinetic studies that validate this approach (36). RDV is available in two formulations, injectable solution and lyophilized powder, the latter preferred in children due to lower risk of renal impairment. RDV is contraindicated in renal insufficiency with eFGR <30 mL/min (in case of eFGR > 30 mL/min no dose adjustment is required) and hypertransaminasemia (ALT ≥ 5x). Before starting therapy renal and liver function tests are required and interaction with other drugs, such as hydroxychloroquine, azithromycin or antiepileptic drugs, should be considered because of increased risk of hepatocellular toxicity. During therapy, daily monitoring of liver function tests is therefore indicated (25, 36, 47). Moreover, RDV cardiovascular side effects are known in adults (48) and a case of asymptomatic sinus bradycardia was reported in a 13-year-old boy. However, limited data are available regarding the occurrence of arrhythmias in children with COVID-19 and the causal correlation between RDV and bradycardia cannot be established (49). In general, electrocardiographic monitoring is suggested in all admitted children with COVID-19, especially if they are receiving RDV (50). Finally, hypersensitivity reactions are reported (36).

### Immunomodulating Therapy

Immune-mediated lung injury and systemic hyperinflammation are characteristic of severe COVID-19 in adults. Even in some children SARS-CoV-2 appears to trigger a dysregulated hyperinflammatory process that may benefit from immunomodulation (24). While numerous trials of immunomodulatory therapies for COVID-19 in adults are launched, few clinical trials in children are currently enrolling, therefore no guidance can be provided (39). Immunomodulating therapy in children should be considered, on an individual basis, in severe cases with PARDS or in presence of progressive deterioration of respiratory function or alteration of inflammatory markers (IL-6, D-dimer, ferritin and C-reactive protein) (25). Since an early strong immune response is required for the effective clearance of the virus, immunomodulating therapies should be administered at least 7 days from the beginning of the symptoms, when exaggerated host immune response could appear (25, 32). Non-specific immunomodulators include corticosteroids and intravenous human immunoglobulin (IVIG). Systemic corticosteroids, repressing pro-inflammatory genes transcription and inhibiting cytokines production, are effective in many inflammatory conditions (32, 39). Liu et al. (51, 52) showed that short-term therapy with low dose corticosteroids (≤ 2 mg/kg/day) in adults with COVID-19 was effective in the inhibition of IL-6 production, improving cytokine storm control. In a retrospective study on Chinese patients with ARDS, treatment with methylprednisolone was associated with mortality reduction (5). In addition, the multicenter Randomized Evaluation of COVID-19 Therapy (RECOVERY) showed a reduction in 28-day mortality among adults treated with dexamethasone who were receiving respiratory support (53). Thus, corticosteroids should be considered also in children with ARDS or respiratory deterioration, at least 7 days after onset of infection, in order to reduce the risks of prolonged viral shedding and secondary bacterial infections (25, 39, 54, 55). IV methylprednisolone is recommended at a dosage not exceeding 1–2 mg/kg/day (max 80 mg/day) for 3–5 days or higher dose with 30 mg/kg/day bolus in severe cases (54). Alternatively, oral dexamethasone 0.2–0.4 mg/kg (max 6 mg/day) for 10 days could be considered (25, 26). Unlike corticosteroids, the use of intravenous immunoglobulin (IVIG) for treatment of lung disease in COVID-19 pediatric patients is not recommended, because of the lack of proven efficacy (39). In specific clinical scenarios, such as severe thrombocytopenia associated with COVID-19, IVIG could be considered (39). A recent study shows that IVIG lots available from the USA and other parts of the world may variably contain antibodies to SARS-CoV-2, but future clinical trials are needed to demonstrate their neutralizing power (39, 56). In critical COVID-19 pediatric patients with high levels of IL-6/D-dimer/PCR/ferritin/fibrinogen, the use of specific immunomodulators directed against proinflammatory cytokines could be considered. These drugs should be administered with the right timing, at the end of the initial phase of high viral load of COVID-19 (afebrile > 72 h and/or at least 7 days after the onset of symptoms), when cytokine storm develops (25, 30, 32, 39, 55, 57). Since IL-6 plays an important role in COVID-19 “cytokine storm” and elevated levels are predictive of a fatal outcome (24, 51, 52, 58), tocilizumab (IL-6 receptor monoclonal antibody) has been proposed to treat critical patients (59). Tocilizumab treatment was associated with decreased risk of mechanical ventilation or death in several cohort studies of adult COVID-19 patients (60, 61). Dosing for tocilizumab in children (12 mg/kg IV in <30 kg, 8 mg/kg IV in >30 kg, max 800 mg/dose) is based on use for rheumatologic indications. In case of no response, a second infusion may be considered after 12 h and a third infusion after 24 h (25, 39). Another specific immunomodulator, anakinra (human IL-1 receptor antagonist), was reported to reduce both need for invasive mechanical ventilation and mortality in several adult case reports (62, 63). The extrapolation of these data, in association with pediatric case reports (64, 65), may justify its use in children with severe pulmonary involvement (25). Anakinra can be administered intravenously (off label) or subcutaneously, at the dosage of 8–10 mg/kg/day in 2-4 administrations (max 100 mg 4 times/day) (25, 59).

### Passive Immunization: Convalescent Plasma Treatment

In the absence of specific antivirals, the transfusion of hyperimmune SARS-CoV-2 convalescent plasma has emerged as a promising therapeutic option. CPT primarily acts through viral neutralization, although other mechanisms such as stimulation of antibody-dependent cellular cytotoxicity and enhanced phagocytosis have been posited (66). However, a recent randomized clinical trial in adults showed no significant improvement of CPT in the clinical course (67). In children, data of efficacy are limited to case series without matched controls (32, 68). These reports suggest that CPT may be of greatest benefit in early disease course before generation of endogenous antibodies or in immunocompromised patients (69), but has theoretical risks (allergic reaction, transfusion-associated circulatory overload, infection, antibody-dependent enhancement). CPT was tested at the Children's Hospital of Philadelphia in four PARDS cases which demonstrated safety and possible efficacy (70). Currently, CPT (200–500 mL in children >40 kg, 10–15 mL/kg in children <40 kg) may be considered in severe pediatric COVID-19 as part of a clinical trial (39). However, international clinical trials are needed to prove CPT efficacy in adults and children (25, 39, 71).

### Venous Thromboembolism Prophylaxis

In adults severe COVID-19 seems to be associated with an increased thrombotic risk, resulting from an inflammatory-driven endothelial dysfunction and hypercoagulable state (25, 72). A study on 449 adults with severe infection showed a lower mortality rate in those receiving anticoagulant therapy (73). Therefore, adult protocols suggest prophylactic administration of low molecular weight heparin (LMWH), especially in case of ARDS (74). However, children with COVID-19 have a lower incidence of thrombotic complications than adults (25). An Italian observational study recorded a prevalence of 1 venous thromboembolism (VTE) in more than 350 hospitalized pediatric COVID-19 cases, compared to an estimated incidence of hospital-acquired VTE in the general pediatric population of ~1/200 (72). Moreover, Duarte-Salles et al. (75) reported a bleeding rate of 2–3% in hospitalized children with COVID-19. Thus, contrary to adult guidance, universal anticoagulant prophylaxis in hospitalized children suffering from COVID-19 is not advised, but a careful balance of thrombotic and hemorrhagic risks for every child should be assessed (72). Anticoagulant thromboprophylaxis should be administered in hospitalized children with COVID-19–related illness (including MIS-C) who have superimposed risk factors for hospital-associated VTE (Table 2) (76). In clinically stable children without severe renal impairment low-dose LMWH subcutaneously twice daily, targeted to achieve a 4-h post-dose anti-Xa activity level of 0.2 to <0.5 U/mL, is the preferable option; for children clinically unstable or with renal impairment unfractionated heparin is suggested by continuous intravenous infusion, targeting an anti-Xa activity of 0.1 to <0.35 U/mL (76).

**Table 2:** Risks factors for hospital VTE in children [adapted from Goldenberg NA et al. (76)].

**•Central venous catheter**
**•Mechanical ventilation**
**•Prolonged length of stay**
**•Complete immobility**
**•Obesity**
**•Active malignancy, nephrotic syndrome, cystic fibrosis exacerbation, sickle cell disease vaso-occlusive crisis, or flare of underlying inflammatory disease (e.g., lupus, juvenile idiopathic arthritis, inflammatory bowel disease)**
**•Congenital or acquired cardiac disease with venous stasis or impaired venous return**
**•Previous history of VTE or first-degree family history of VTE before age 40 years or unprovoked VTE**
**•Known thrombophilia (e.g.,: protein S, protein C, or antithrombin deficiency; factor V Leiden; factor II G20210A; persistent antiphospholipid antibodies)**
**•Pubertal, post-pubertal, or age > 12 years**
**•Receiving estrogen-containing oral contraceptive pill**
**•Status-post splenectomy for underlying hemoglobinopathy**

### Antibiotic Therapy

Langford et al. (77) showed that three-quarters of patients with COVID-19 receive antibiotics and prescription is higher than the estimated prevalence of bacterial coinfection (8.6%), thus resulting in increased selective pressure for antimicrobial resistance. Routine antibiotics and antifungal drugs must be avoided and used only when coinfections are proven or strongly suspected (2, 30, 78) or in patients presenting comorbidities (BDP, CF, immunodeficiency, neuromuscular pathologies). Some authors suggest the use of azithromycin (15 mg/kg/day orally on the first day, followed by 7, 5 mg/kg/day for 4 days) for its anti-inflammatory and antiviral activity demonstrated *in vitro* against Zika and Ebola (25, 79). However, azithromycin has been used in critically ill adults and data on efficacy and safety have not been reported yet, thus caution should be used also in children (2).

### Respiratory Support

SARS-CoV-2 in children can lead to serious respiratory manifestations up to PARDS, although respiratory failure is described only in a small percentage of infected children. In a metanalysis including 1,117 patients <18 years, 2.1% suffered from severe respiratory involvement and 1.2% presented a critical evolution, requiring non-invasive ventilation (1 case) or mechanical ventilation (8 cases) (80). The rate of severe respiratory involvement was slightly higher in a European multicenter cohort study on 582 children, in which 13% required oxygen support, 5% were treated with continuous positive airway pressure (CPAP) and 4% needed mechanical ventilation (81). Unfortunately, details regarding type, timing and parameters of respiratory support required by children with COVID-19 severe pulmonary involvement remain scarce and not detailed. Most published data are based on adults, and cannot always be applied to children, while many other studies report the standard approach used in children with ARDS of different etiology. Type 1 or hypoxemic respiratory failure is the hallmark of SARS-CoV-2 pneumonia and it is primarily caused by intrapulmonary shunting, downregulation of ACE2 expression leading to increased release of pro-inflammatory cytokines and vascular permeability, and lung capillary endothelial injuries causing intravascular microthrombi. With the progression of the disease, the basement membrane is covered by fibrin, cells debris and complement activation products, also resulting in an impairment of lung diffusion capacity (DLCO) (82, 83). Therefore, the characterization of the degree of respiratory failure, in addition to monitoring respiratory drive and effort, are essential to guide the appropriate respiratory support in those children.

#### Supplemental Oxygen

Low flow oxygen represents the first therapeutic option in children with mild respiratory distress, normo-hypocapnia and peripheral oxygen saturation (SpO_2_) ≤ 92% (84). Oxygen should be started with the lowest flow titrated to target SpO_2_ ≥ 92–96% (6, 85) and can be administered through different devices (nasal cannula, face mask with/without reservoir or Venturi mask), depending on the patient tolerability and the fraction of inspired oxygen (FiO_2_) that each device can deliver (86). In case of mild-to-moderate hypoxemia and dyspnea non-responsive to low-flow oxygen, the therapeutic approach should involve the use of High Flow Nasal Cannula (HFNC), which combines the improvement of alveolar gas exchange with the reduction of respiratory work. The flow rate of 1.5–2 L/kg/min, suggested in pediatric practice, generates a positive end-expiratory pressure (PEEP) of approximately 4–6 cmH_2_O, which provides distending pressure of the lung, decreasing the work of breathing (87). Anyway, children on HFNC should be closely monitored for possible respiratory deterioration and in case of no response within 60 min, a rapid escalation to non-invasive respiratory support should be considered (85).

#### Non-invasive Ventilation

In case of impaired gas exchange despite HFNC, the introduction of Non-invasive Ventilation (NIV) is mandatory to improve alveolar recruitment, decrease the ventilation-perfusion mismatch, increase end-expiratory lung volume and support respiratory muscle, reducing the work of breathing. CPAP is the treatment of choice in case of severe hypoxemic normocapnic respiratory insufficiency, providing continuous, monitorable and adjustable PEEP, and it is commonly delivered using nasal mask or helmet. In children it is suggested to begin with a pressure of 5 cmH_2_O, subsequently adapting the pressure as clinically needed (87). However, in case of respiratory exhaustion with increased PaCO_2_, it's mandatory to associate PEEP with inspiratory pressure support (NIV bi-level) to relieve respiratory muscles fatigue, favoring CO_2_ elimination and avoiding invasive ventilation. In bi-level mode, starting with low inspiratory (IPAP 8–12 cmH_2_O) and expiratory (EPAP 4–6 cmH_2_O) pressure could be prudent in order to minimize risk of barotrauma, especially in the youngest; parameters can be adjusted based on respiratory efforts, oxygenation (SpO_2_ target ≥ 92%) and PaCO_2_. In presence of severe respiratory failure associated with PARDS (PaO_2_/FiO_2_ <300) unresponsive to NIV, invasive ventilation is mandatory (6, 26). Like HFNC, NIV is aerosol-generating with environmental dispersion of the virus, therefore adoption of some precautions, such as the use of helmet in CPAP setting or a double circuit (inspiratory-expiratory) and specific antibacterial/viral filters in expiratory circuit in NIV bi-level is advocated (26). In critically ill patients with refractory hypoxemia (SpO_2_ <90%, FiO_2_ > 60%) and hypercapnia unresponsive to ventilation, inhaled nitric oxide and recruitment maneuvers with PEEP cycles to reduce ventilation/perfusion mismatch have been described in some case reports (6). Finally, in patients with increased respiratory efforts and respiratory acidosis with PaO_2_/FiO_2_ <50 for 3 h or 50–80 for 6 h, veno-venous Extracorporeal Membrane Oxygenation is an extreme therapeutic chance (26).

#### Prone Positioning

Prone positioning, reducing pleural pressure gradient and improving alveolar recruitment in the dorsal areas of the lung, has been shown to improve oxygenation and to optimize the benefits of PEEP ventilation in adults with severe ARDS (88). In children with respiratory failure, prone positioning seems to provide a more homogenous alveolar recruitment with higher PEEP, resulting in a better ventilation (88). However, studies evaluating this strategy in children with ARDS are limited and fail to demonstrate a clear and prolonged benefit (89). Moreover, this approach seems to be an efficacious supportive strategy in adults when patients are kept in prone position for at least 12 h a day for many days (90) and could be challenging in awake and dyspnoic pediatric patients. Hence, the use of prone position can be considered a therapeutic option only in intubated children with severe respiratory failure (91).

### Sperimental Strategies

The growing knowledge of COVID-19 pathophysiology and the increasing development of biological drugs are the starting point for future research. In November 2020 the FDA authorized the use of two monoclonal antibodies (casirivimab and imdevimab) able to bind SARS-CoV-2 protein S, preventing cell penetration. These drugs are currently reserved for adults and adolescents >12 years at risk for severe disease (71). However, the main effort is focused on modulation of cytokine storm, instead of control of the primary infection. IFN-γ signaling has been demonstrated to be important in the pathophysiology of COVID-19 (24). There are ongoing adult trials with emapalumab, a monoclonal antibody directed against IFN-γ, approved for treating pediatric and adult patients with primary hemophagocytic lymphohistiocytosis (32). Since complement plays a role in ARDS, another therapeutic approach could be based on complement inhibitors. There are ongoing trials of C5-specific antibody eculizumab on adults with severe COVID-19 (29).

Finally some papers showed that the spike protein by itself (without being part of the corona virus) can damage endothelial cells and disrupt the blood-brain barrier (92). Nutraceuticals with anti-oxidant and anti-viral properties, such as liposomal blend of the natural flavonoids luteolin and quercetin, could prevent the detrimental actions of spike protein, but further case-control studies in pediatric setting are needed (92–94).

## Conclusions

Currently, there is no evidence on the best therapeutic approach for COVID-19 lung disease in children and data are mostly derived from clinical trials conducted in adults. The approach tends to be conservative and based mostly on supportive therapy. However, in hospitalized children with critical illness and worsening lung function, antiviral therapy with remdesivir and immunomodulant treatment could be considered in association to respiratory support.

## Author Contributions

EG and MPi conceptualized the paper, reviewed full-text articles, extracted the data, and wrote the first draft of the manuscript. Searches and screening of papers were done by ML, MPe, BM, LC, and VH, who were advised by MPa. MPa, SB, SA, and PM helped to revise the paper and consider policy implications. All authors contributed to revision of the final version of the manuscript.

## Conflict of Interest

The authors declare that the research was conducted in the absence of any commercial or financial relationships that could be construed as a potential conflict of interest.

## Publisher's Note

All claims expressed in this article are solely those of the authors and do not necessarily represent those of their affiliated organizations, or those of the publisher, the editors and the reviewers. Any product that may be evaluated in this article, or claim that may be made by its manufacturer, is not guaranteed or endorsed by the publisher.

## Abbreviations

SARS-CoV-2: severe acute respiratory syndrome coronavirus 2
MIS-C: multisystem inflammatory syndrome in children
HCoVs: human coronavirus
ARDS: acute respiratory distress syndrome
PARDS: pediatric acute respiratory distress syndrome
ACE-2: angiotensin converting enzyme-2 receptor
IS: immune system
CF: cystic fibrosis
BDP: bronchodysplasia
IV: intravenous
LPV/r: lopinavir/ritonavir
RDV: remdesivir
IVIG: intravenous human immunoglobulin
CPT: convalescent plasma treatment
LMWH: low molecular weight heparin
VTE: venous thromboembolism
CPAP: continuous positive airway pressure
DLCO: lung diffusion capacity
SpO2: peripheral oxygen saturation
FiO2: fraction of inspired oxygen
HFNC: high flow nasal cannula
PEEP: positive end-expiratory pressure
NIV: non-invasive ventilation
IPAP: inspiratory positive airway pressure
EPAP: expiratory positive airway pressure.
